# Supplementary material for: JAZF1 safeguards human endometrial stromal cells survival and decidualization by repressing the transcription of G0S2
Source: Commun Biol. 2023 May 27;6:568. doi: 10.1038/s42003-023-04931-x (PMC10224957; doi:10.1038/s42003-023-04931-x)

Supplementary Table 1. Information on decidua samples for western blotting

	Normal pregnancy (n=10)	RSA pregnancy (n=10)	p
Age	30.40±3.950	31.70±2.003	>0.05
Gestational week	6.500±0.7071	7.200±0.9189	>0.05
Gravidity	3.700±1.337	4.500±2.550	>0.05
Parity	1.500±0.8498	0.3000±0.4830	<0.05
Miscarriage times	0.2000±0.4216	3.600±2.797	<0.05

Supplementary Table 2. Information on decidua samples for RT-qPCR

	Normal pregnancy (n=19)	RSA pregnancy (n=20)	p
Age	29.42±4.363	31.25±4.141	>0.05
Gestational week	6.947±1.026	7.000±1.338	>0.05
Gravidity	3.737±1.558	3.550±2.064	>0.05
Parity	1.526±1.073	0.2000±0.4104	<0.05
Miscarriage times	0.1579±0.3746	2.850±2.084	<0.05

Supplementary Table 3. Information on DSCs isolated from decidua for western blotting

	Normal pregnancy (n=6)	RSA pregnancy (n=5)	p
Age	31.17±2.787	30.20±3.493	>0.05
Gestational week	6.167±0.4082	6.200±0.4472	>0.05
Gravidity	1.833±0.7528	3.000±0.7071	<0.05
Parity	1.667±0.8165	0.2000±0.4472	<0.05
Miscarriage times	0.1667±0.4082	3.200±1.304	<0.05

Supplementary Table 4. Information on DSCs isolated from decidua for RT-qPCR

	Normal pregnancy (n=3)	RSA pregnancy (n=3)	p
Age	30.33±3.215	30.00±3.464	>0.05

Gestational week	6.000±0.000	6.333±0.5774	>0.05
Gravidity	1.667±0.5774	3.000±1.000	>0.05
Parity	1.333±0.5774	0.3333±0.5774	>0.05
Miscarriage times	0.3333±0.5774	3.000±1.000	<0.05

Supplementary Table 5. Information on ESCs isolated from endometrium for cell models

Number	#2	#3
Age	32	27
Infertility years	3	2
Diagnosis	Primary infertility (tubal factors)	Primary infertility (tubal factors)
Gravidity	0	0
Parity	0	0
Miscarriage times	0	0

Supplementary Table 6 All primers used in this study

	**Forward primer (5'-3')**	**Reverse primer (5'-3')**
**CRISPR/Cas9 sgRNA**		
JAZF1: sgRNA	CACCGTGTCCAGCTCAGTGTCTCGA	AAACTCGAGACACTGAGCTGGACAC
**DNA PCR primer**		
JAZF1	TAGAAGATAATTCCATTGCA	ACTAGTGAAAGGATCAACGA
**siRNA sequence**		
siRNA sequence-*JAZF1*	CCACAGCAGUGGAAGCCUUTT	AAGGCUUCCACUGCUGUGGTT
siRNA sequence-*G0S2*	CCAACACUGUGUGAAUUAUTT	AUAAUUCACACAGUGUUGGTT
siRNA sequence-*Purβ*	GCCGGUAUGCGGAUGAGAUTT	AUCUCAUCCGCAUACCGGCTT
**Overexpression primer**		
JAZF1	CATGACAGGCATCGCCGCCGCCT	CTTATTGCTGCATCTTCCTGATAAT
G0S2	CATGGAAACGGTCCAGGAGCT	CCTAGGAGGCGTGCTGCCGGT
Purβ	TATGGCGGACGGCGACAGC	CTCAATCCTCATCCACCTCCTCACCCT
**Luciferase reporter assay primer**		
G0S2	GTCTCTGAGTTACAGAAACACAGGAAATG	ACCGCTTTAGCGTCCGGG
**qPCR primer**		
β-actin	CTACCTCATGAAGATCCTCACCGA	TTCTCCTTAATGTCACGCACGATT
JAZF1	CGCCTCCTTCTTCTCCAATAC	ATGTGGTTGTCCTCGATGTG
BAX	CCCGAGAGGTCTTTTTCCGAG	CCAGCCCATGATGGTTCTGAT
BCL2	GGTGGGGTCATGTGTGTGG	CGGTTCAGGTACTCAGTCATCC
IGFBP1	CTATGATGGCTCGAAGGCTC	TTCTTGTTGCAGTTTGGCAG
PRL	CATCAACAGCTGCCACACTT	CGTTTGGTTTGCTCCTCAAT
G0S2	GCCGTGCCACTAAGGTCATT	GATCAGCTCCTGGACCGTTT
Purβ	ACGGGCTGTTGATCTCACTG	CAGCCCTGCTCCTCTCAAAA

Supplementary Table 7 The plasmids used in this paper

1	pLentiCRISPRv2-Neo-*JAZF1*
2	pLVX- IRES-Neo-*JAZF1*
3	pLVX- IRES-Neo-*G0S2*
4	pLVX- IRES-Neo-*Purβ*
5	pGL3-Basic-*G0S2*
6	pcDNA3.1

Supplementary Table 8 Detailed results of mass spectrometry (upload in excel form, named Supplementary Data 2)

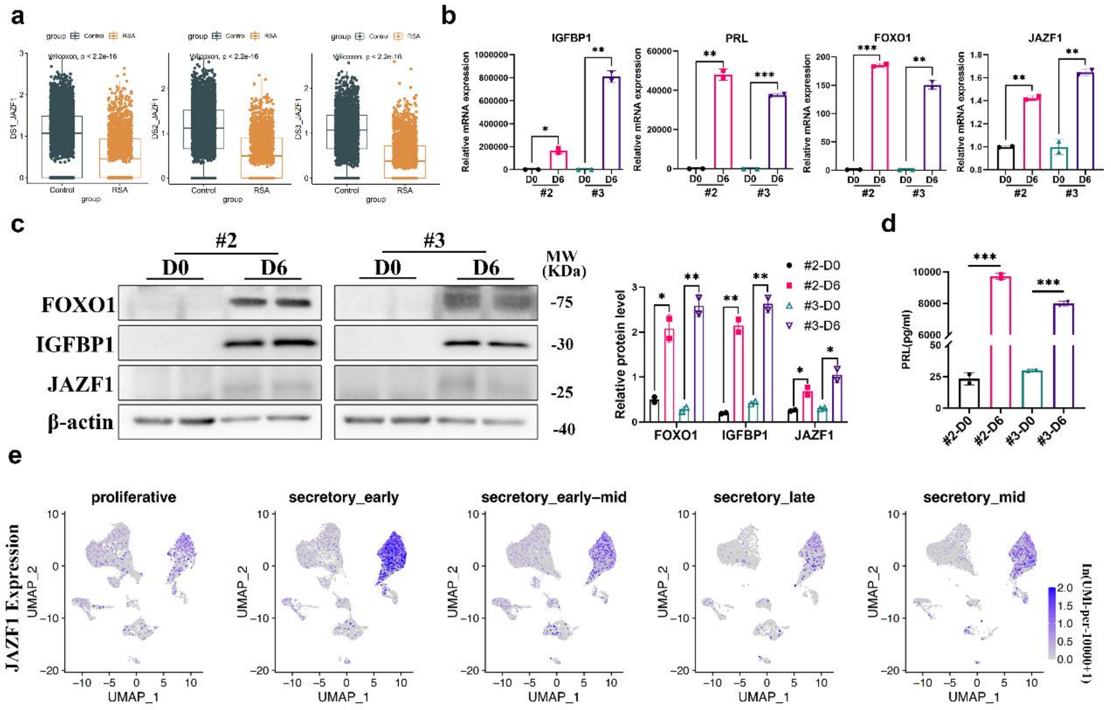

Supplementary Figure 1. Expression of JAZF1 in primary decidualized endometrial stromal cells. (a) scRNA-seq analysis of *JAZF1* expression in DS1, DS2 and DS3. The data were shown as the median and quartile. (b-c) RT-qPCR (b), western blotting (c) analysis of IGFBP1, FOXO1 and JAZF1 in decidualized primary ESCs. (d) ELISA detection of PRL in decidualized primary ESCs. (e) Feature plot of *JAZF1* in proliferative and early-, early- mid-, mid-, and late-secretory phase stromal cells. The data were retrieved from scRNA seq data deposited in the Gene Expression Omnibus (GSE111976). Other data were shown as the mean ± standard deviation (SD). *P<0.05, **P<0.01, ***P<0.001.

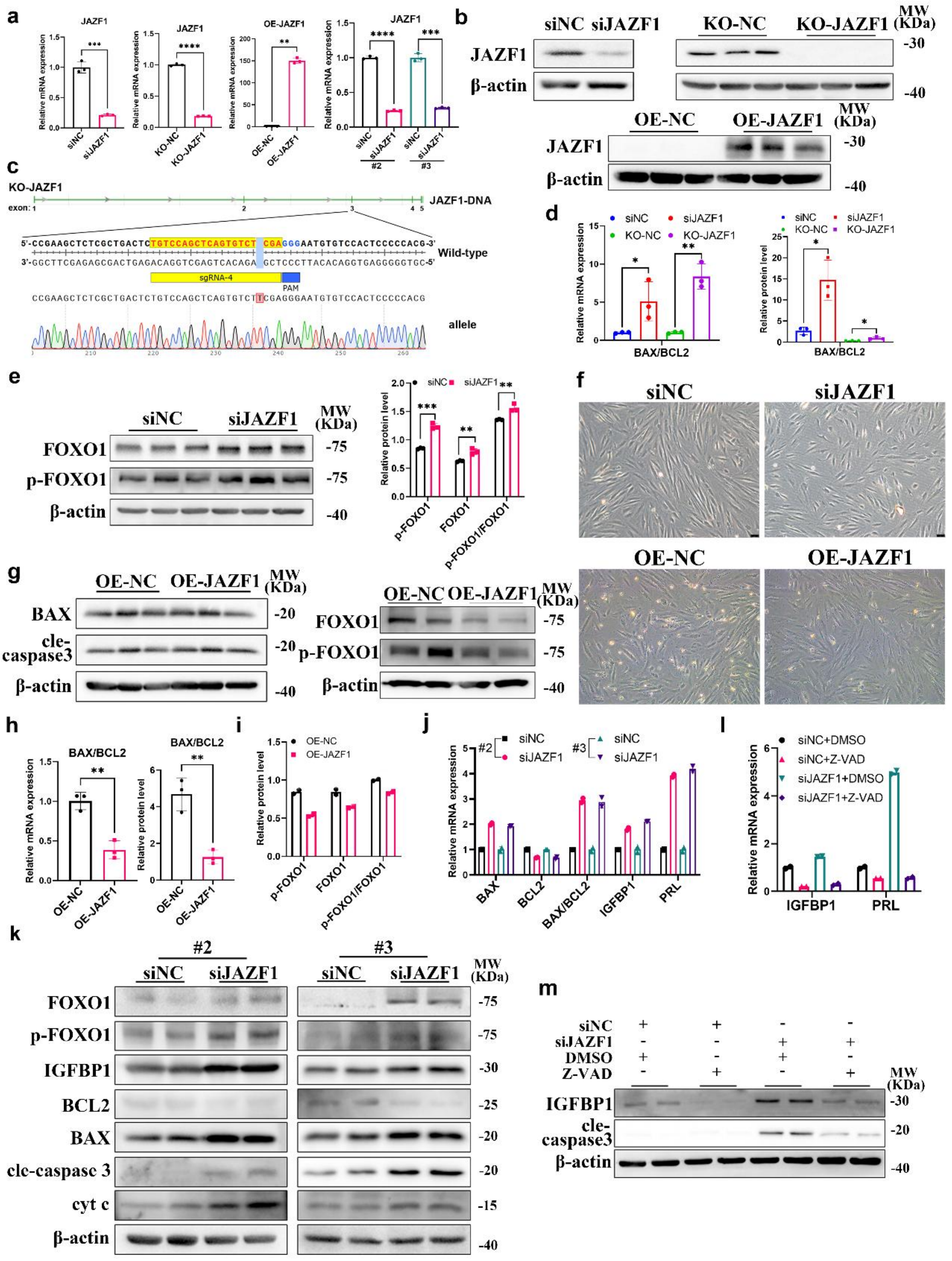

Supplementary Figure 2. Effect of JAZF1 on stromal cells function. The data were shown as the mean ± standard deviation (SD). (a-b) mRNA (a) and protein (b) level of JAZF1 in HESCs and primary ESCs transfected with siRNA or lentivirus. (c) Schematic diagram of the structure of human *JAZF1* gene and its mutant form (KO) targeted by CRISPR/Cas9. (d-e) The expression of BAX/BCL2, FOXO1, p-FOXO1

and p-FOXO1/FOXO1 in decidualized HESCs with JAZF1 depletion. (f) Microscopic images of cell changes 6 days after knockdown or overexpression of JAZF1 with decidualization for 6 days. (g-i) The expression of BAX, cle-caspase3, FOXO1, p-FOXO1 and p-FOXO1/FOXO1 in decidualized HESCs transfected with OE-NC or OE-*JAZF1* lentivirus. (j-l) The expression of decidualization-related and apoptosis-related genes in decidualized primary ESCs with *JAZF1* knockdown. (l-m) The expression of IGFBP1, PRL and cleaved-caspase 3 in decidualized HESCs treated with si*JAZF1* and 20μM Z-VAD-FKM. *P<0.05, **P<0.01, ***P<0.001.

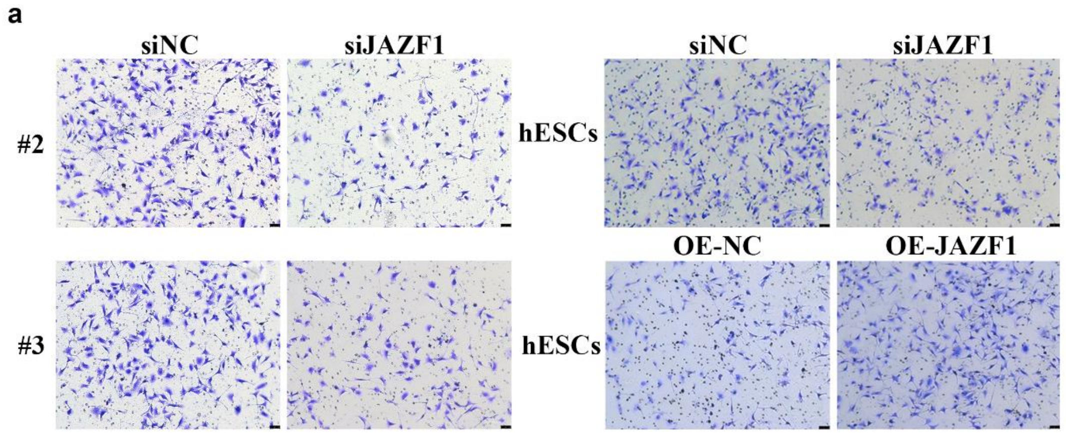

Supplementary Figure 3. Effect of JAZF1 knockdown stromal cells on trophoblast invasion. (a) Transwell demonstrated the effect of the stromal cells with knockdown or overexpression of *JAZF1* on the invasive ability of HTR-8/SVneo. scale bars, 100μm.

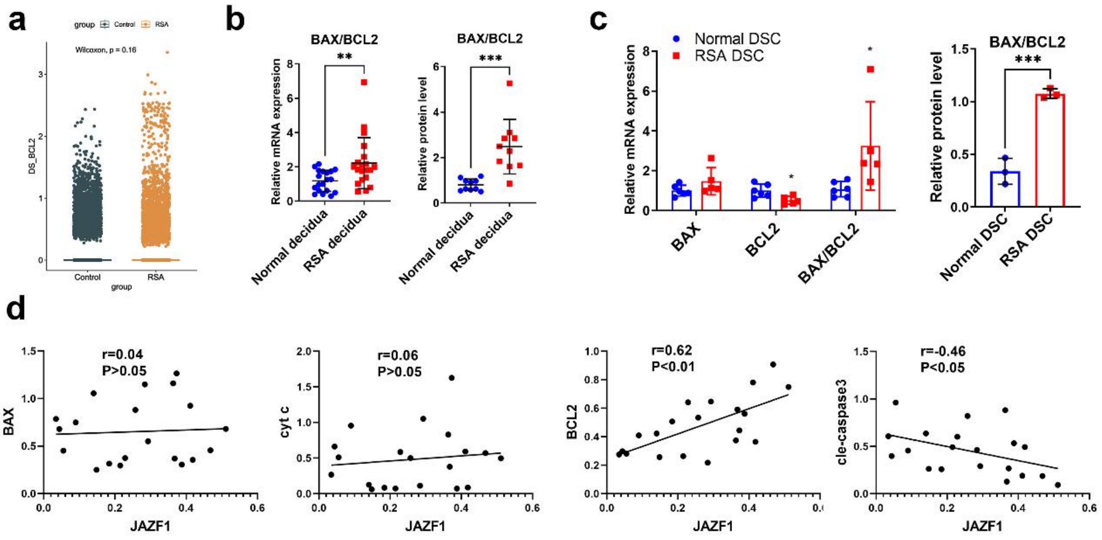

Supplementary Figure 4. Correlation analysis between JAZF1 and apoptosis-related molecules. (a) scRNA-seq analysis of *BCL2* expression in DSCs. identified by single-cell transcriptomics. The data were shown as the median and quartile. (b) The expression of BAX/BCL2 in decidua. (c) The expression level of *BAX*, *BCL2,* BAX/BCL2 in RSA primary DSCs. (d) Correlation analysis of JAZF1 and BAX, BCL2, cyt c, cleaved-caspase 3 protein expression. Other data were shown as the mean ± standard deviation (SD). **P*<0.05, ***P*<0.01, ****P*<0.001.

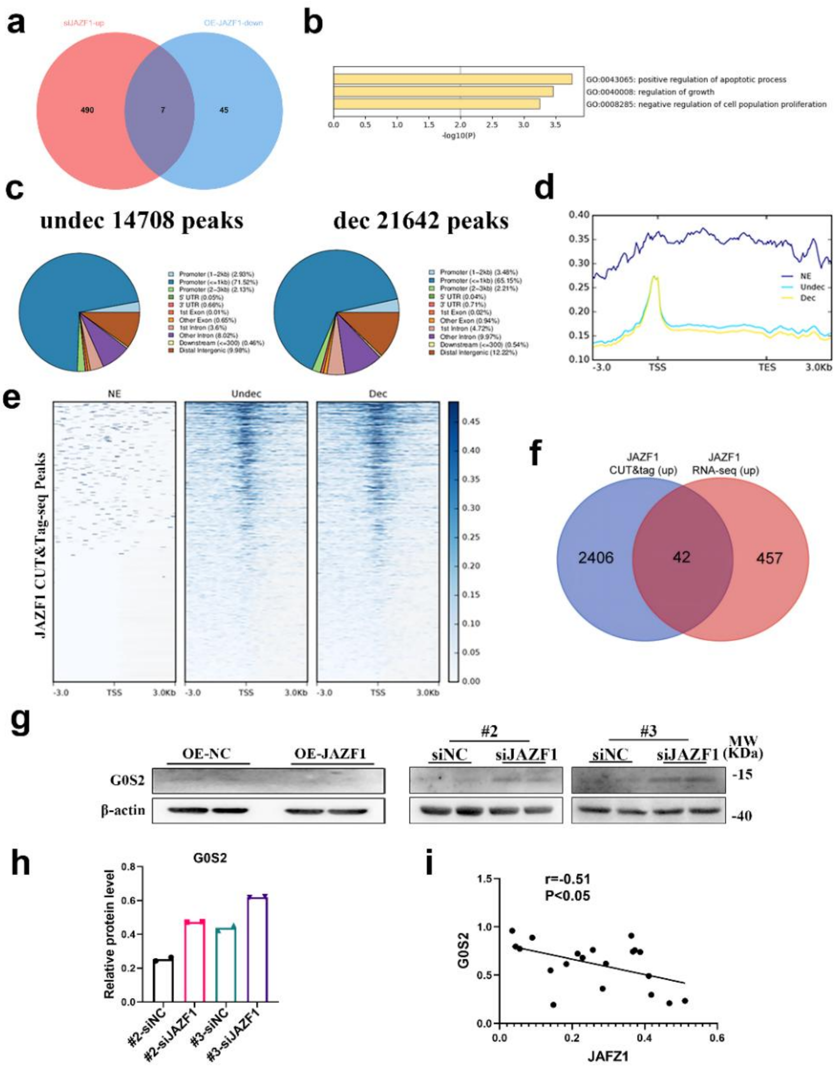

Supplementary Figure 5. Data analysis of JAZF1 RNA-seq and CUT&Tag-seq. The data were shown as the mean ± standard deviation (SD). (a) Venn diagram of JAZF1 upregulated with si*JAZF1* and JAZF1 downregulated genes after *JAZF1* overexpression. (b) GO analysis of overlapped genes of JAZF1 upregulated with si*JAZF1* and JAZF1 downregulated genes after *JAZF1* overexpression. (c) Distribution of JAZF1-binding peaks as revealed from JAZF1 CUT&Tag of undecidualized and decidualized stromal cells. (d) Distribution JAZF1 binding in gene body. (e) Heatmap of JAZF1-binding sites distribution. (f) Venn diagram of JAZF1 binding peaks and JAZF1 upregulated genes after *JAZF1* knockdown. (g-h) Western blotting analysis of G0S2 in HESCs transfected with overexpressing lentivirus and primary ESCs with JAZF1 depletion. (i) Correlation analysis of JAZF1 and G0S2 protein expression. **P*<0.05, ***P*<0.01, ****P*<0.001.

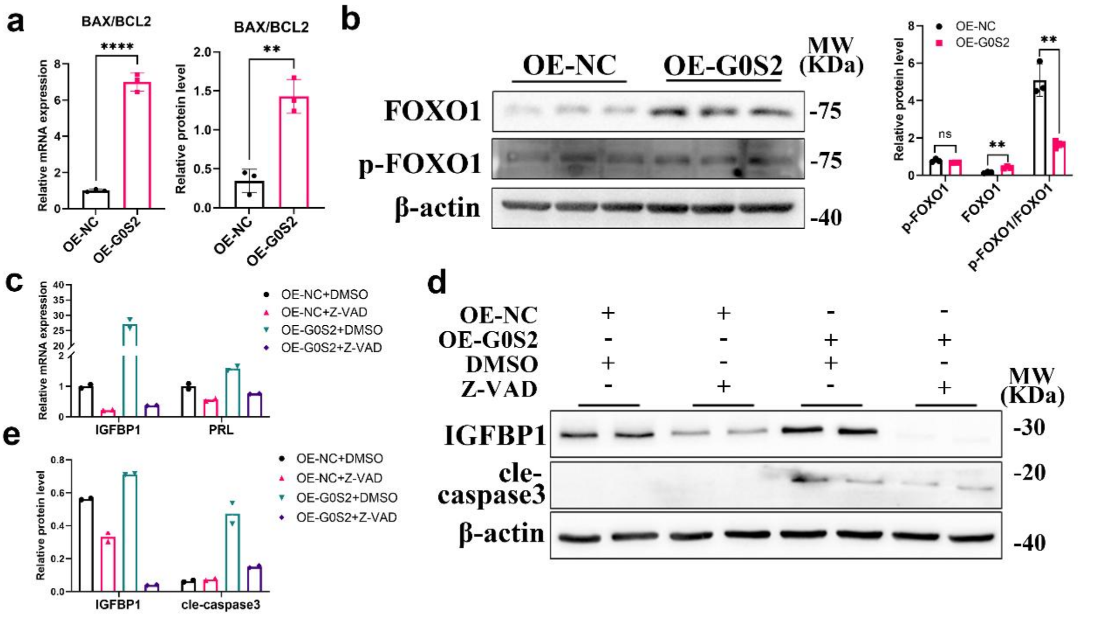

Supplementary Figure 6. Relationship between apoptosis and decidualization defects induced by overexpression of G0S2. The data were shown as the mean ± standard deviation (SD). (a-b) The expression of BAX/BCL2, FOXO1, p-FOXO1, p-FOXO1/FOXO1 in HESCs with *G0S2* overexpression. (c-e) RT-qPCR (c), western blotting (d-e) analysis of IGFBP1, PRL, cleaved-caspase 3 in decidualized OE-*G0S2* treated with 20μM Z-VAD-FKM. n=2. **P*<0.05, ***P*<0.01, ****P*<0.001.

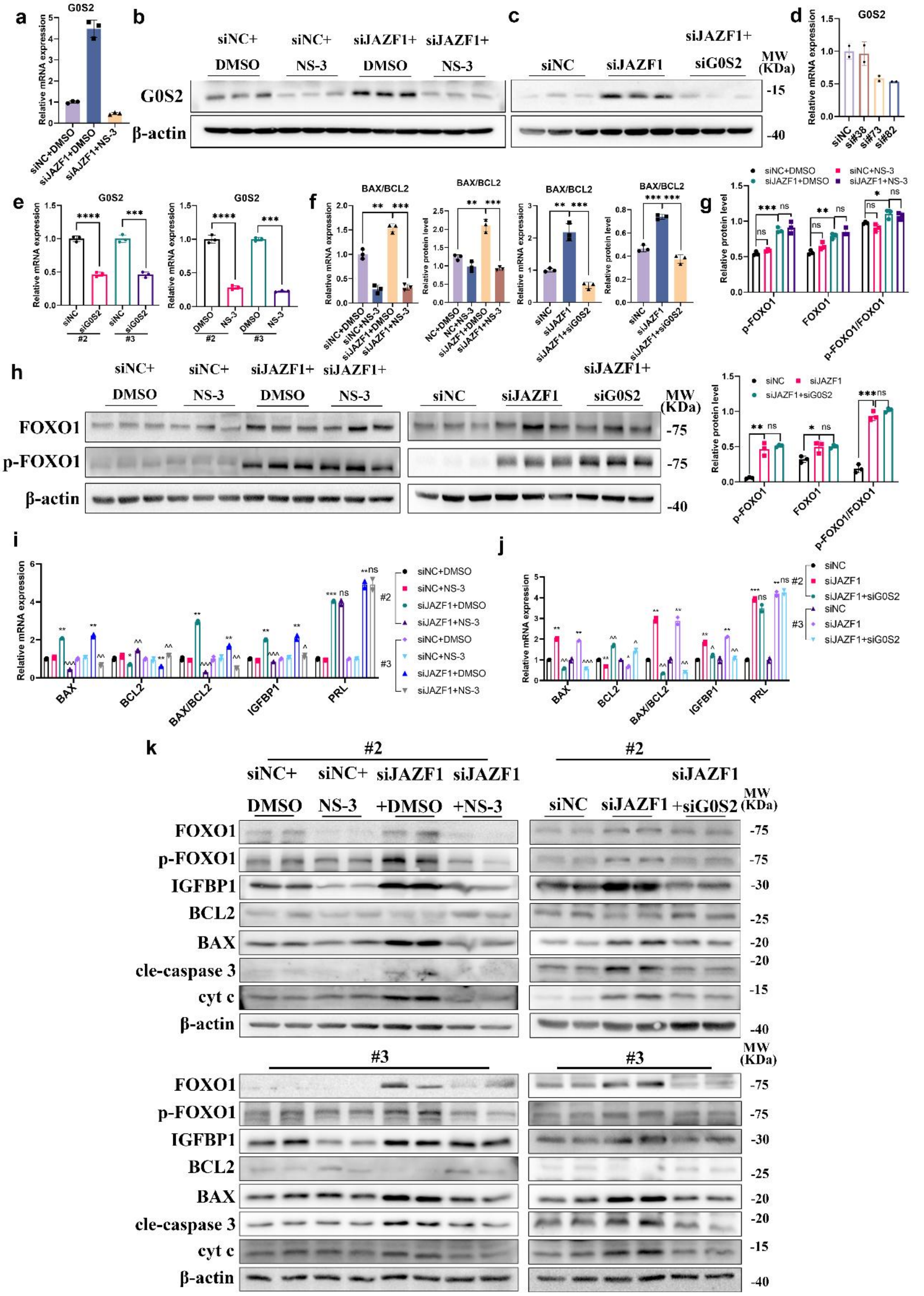

Supplementary Figure 7. JAZF1 depletion promoted cell death by activating G0S2 in primary decidualized endometrial stromal cells. The data were shown as the mean ± standard deviation (SD). (a-b) RT-qPCR (a) and Western blotting (b) expression and analysis for G0S2 in HESCs treated with 22.5μM NS-3. (c-d) mRNA

and protein expression of G0S2 in HESCs transfected with siRNA targeting G0S2. (e) RT-qPCR analysis of *G0S2* in primary ESCs with G0S2 inhibition. (f) The expression of BAX/BCL2 in decidualized HESCs with G0S2 inhibition. (g-h) RT-qPCR (g) and western blotting (h) expression and analysis for FOXO1, p-FOXO1, p-FOXO1/FOXO1 in HESCs with G0S2 inhibition. (i-k) The expression of decidualization-related and apoptosis-related genes in decidualized primary ESCs with G0S2 depletion. *P<0.05, **P<0.01, ***P<0.001.

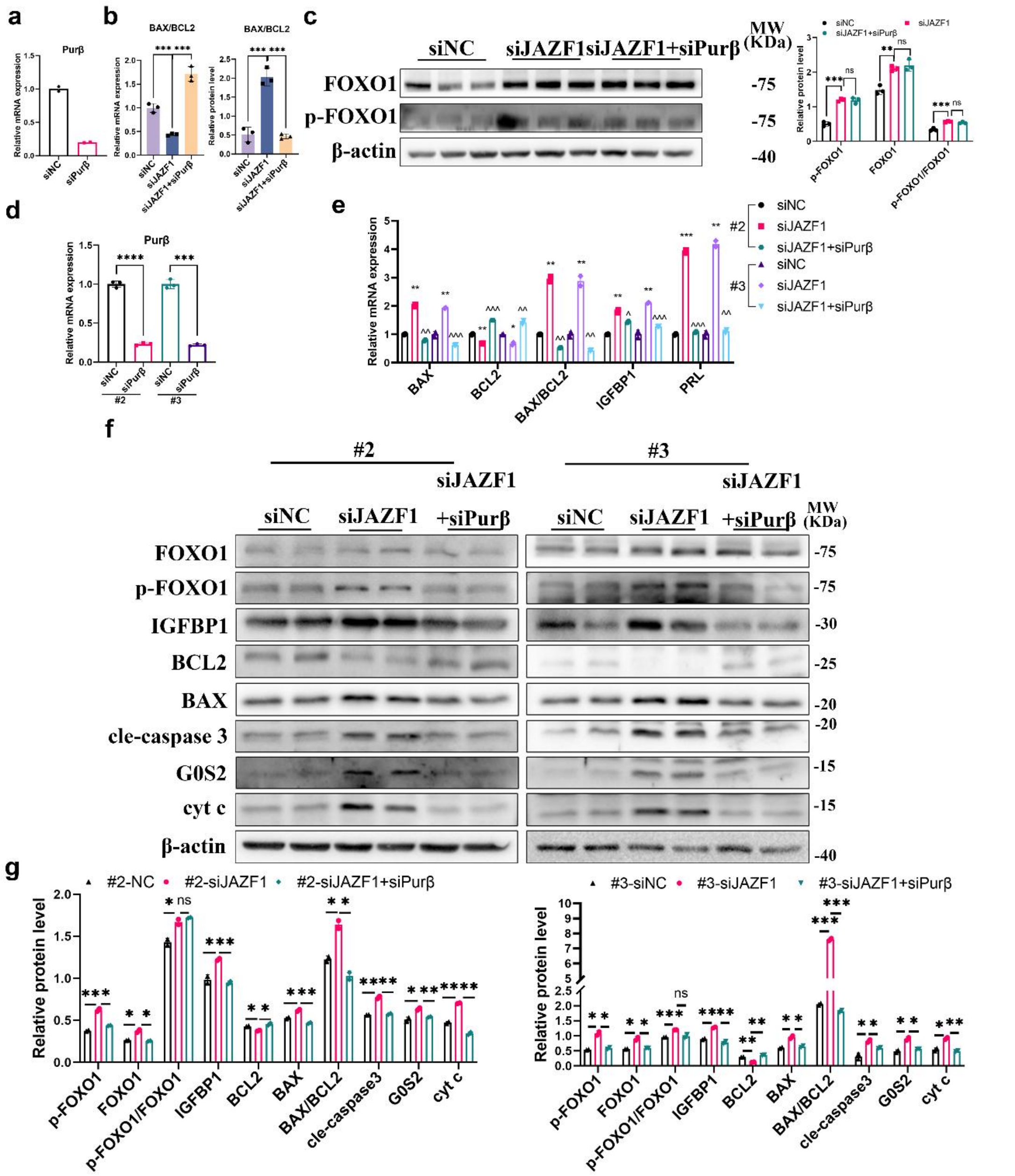

Supplementary Figure 8. JAZF1 depletion promoted cell death by activating G0S2 via Purβ in primary decidualized endometrial stromal cells. The data were shown as the mean ± standard deviation (SD). (a) The efficiency of Purβ in HESCs with *Purβ* knockdown. n=2. (b) The expression of BAX/BCL2 in decidualized HESCs treated with si*JAZF1* and si*Purβ*. (c) Protein expression of FOXO1, p-FOXO1, p-FOXO1/FOXO1 in HESCs transfected with siRNA targeting JAZF1 and Purβ. (d) The efficiency of Purβ in primary ESCs with Purβ knockdown. (e-g) The expression of decidualization-related and apoptosis-related genes in decidualized primary ESCs with JAZF1 and Purβ depletion.

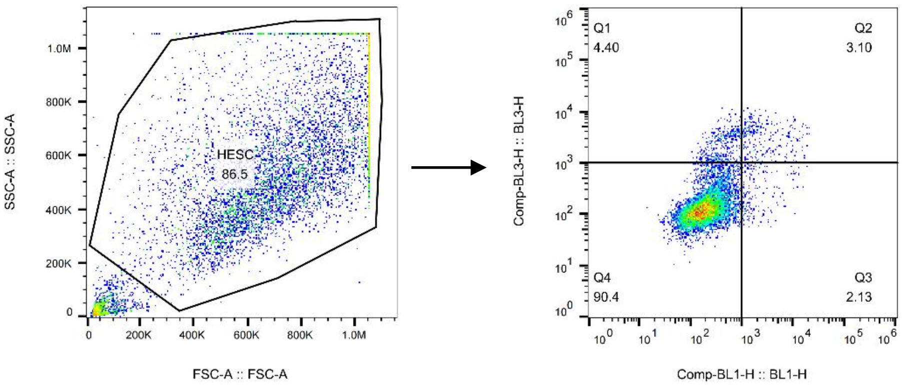

Supplementary Figure 9. Representative flow cytometry gating strategies. Scatterplots of forward scatter vs. side scatter showing live cell gating followed by apoptotic cell partition. BL1-H: Annexin V-FITC; BL3-H: PI.

Supplenentary Figure 10-23 is the original image of the blotting results, with the red box representing cutting from the same membrane. In some images, the results were not combined with the protein ladder, so the ladder pictures are attached separately.

Supplementary Figure 10. Uncropped western blots images for Fig 1.

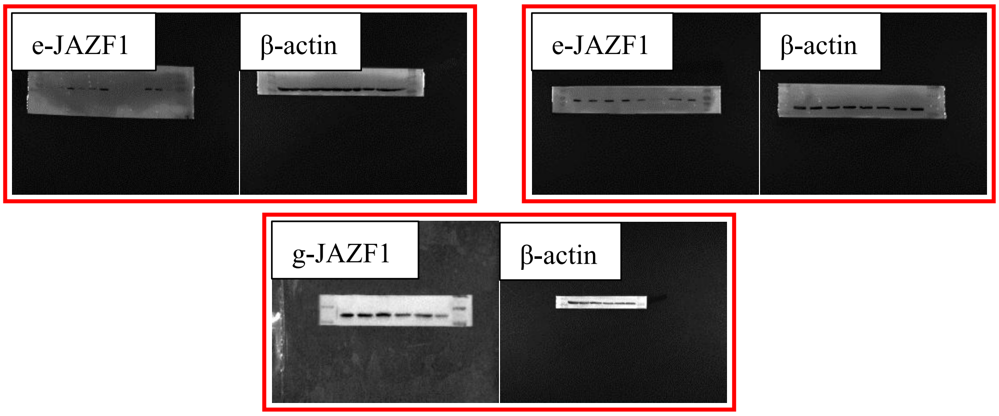

Supplementary Figure 11. Uncropped western blots images for Fig 2

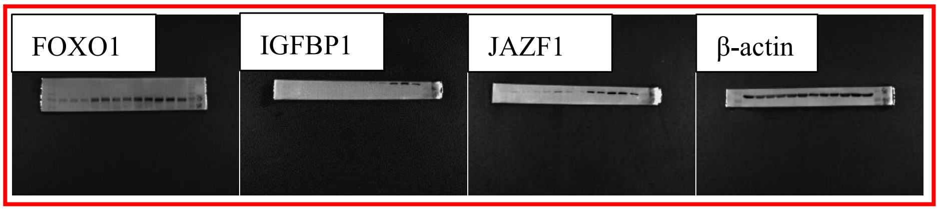

Supplementary Figure 12. Uncropped western blots images for Fig 3

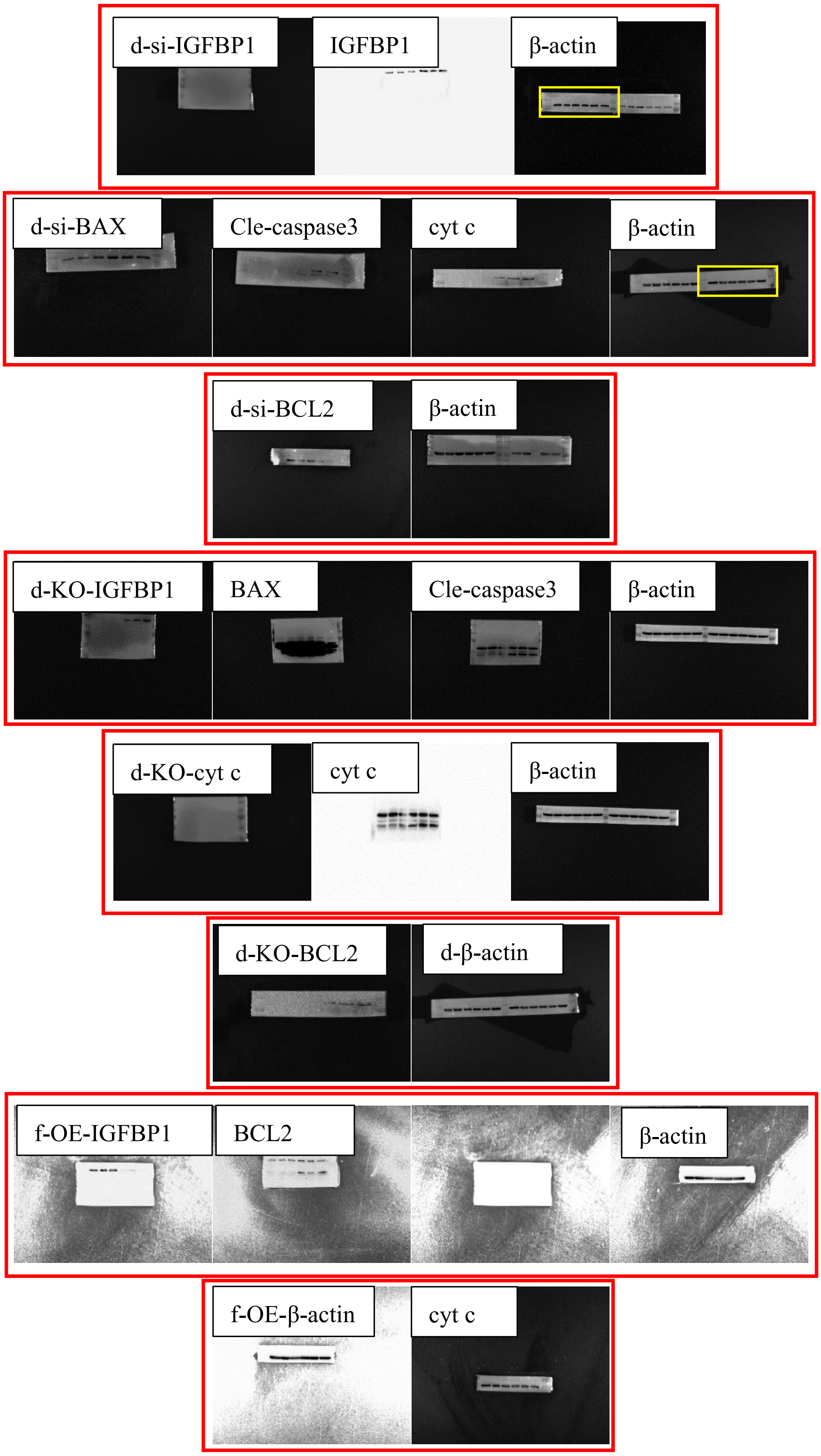

Supplementary Figure 13. Uncropped western blots images for Fig 4

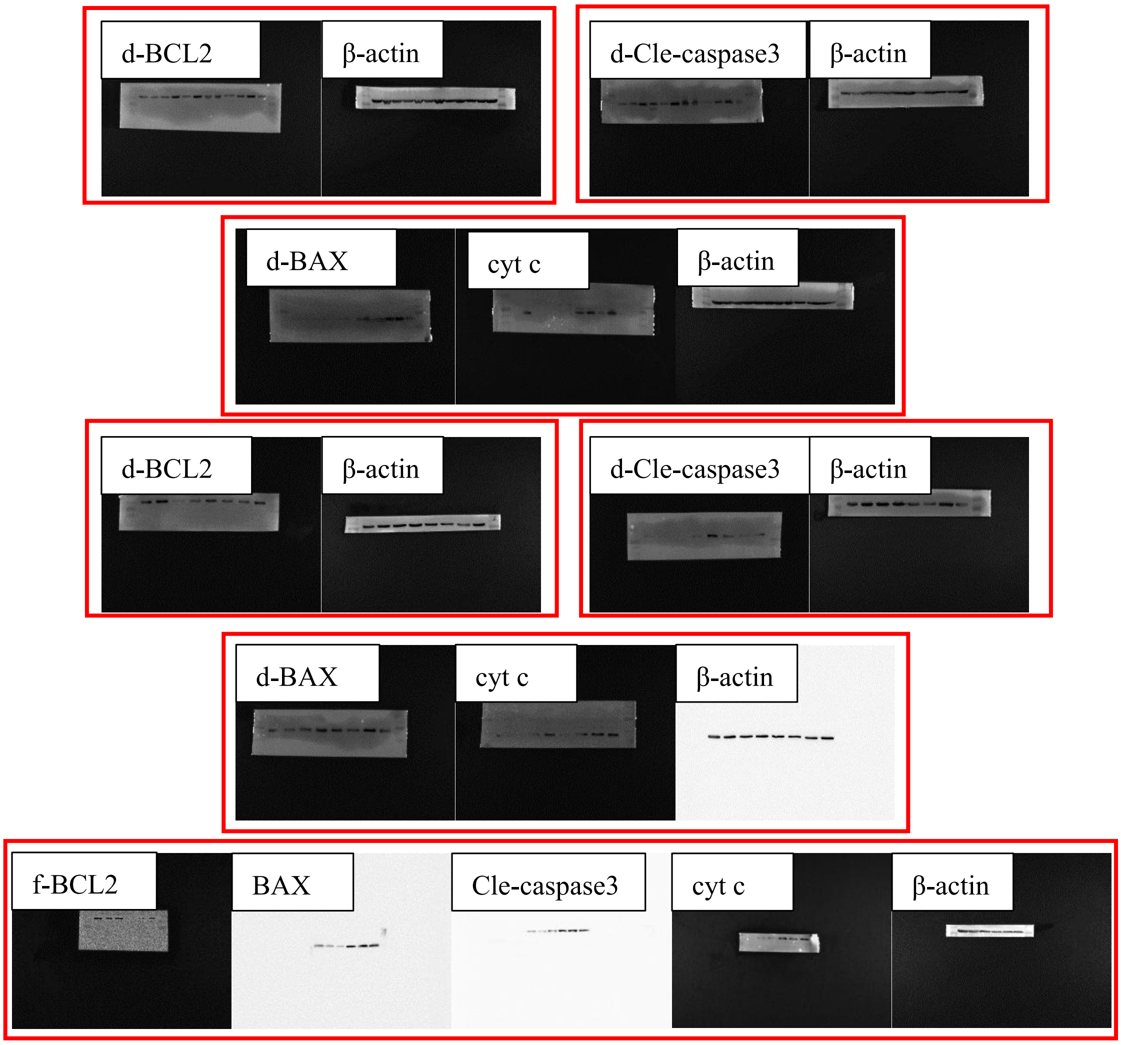

Supplementary Figure 14. Uncropped western blots images for Fig 5

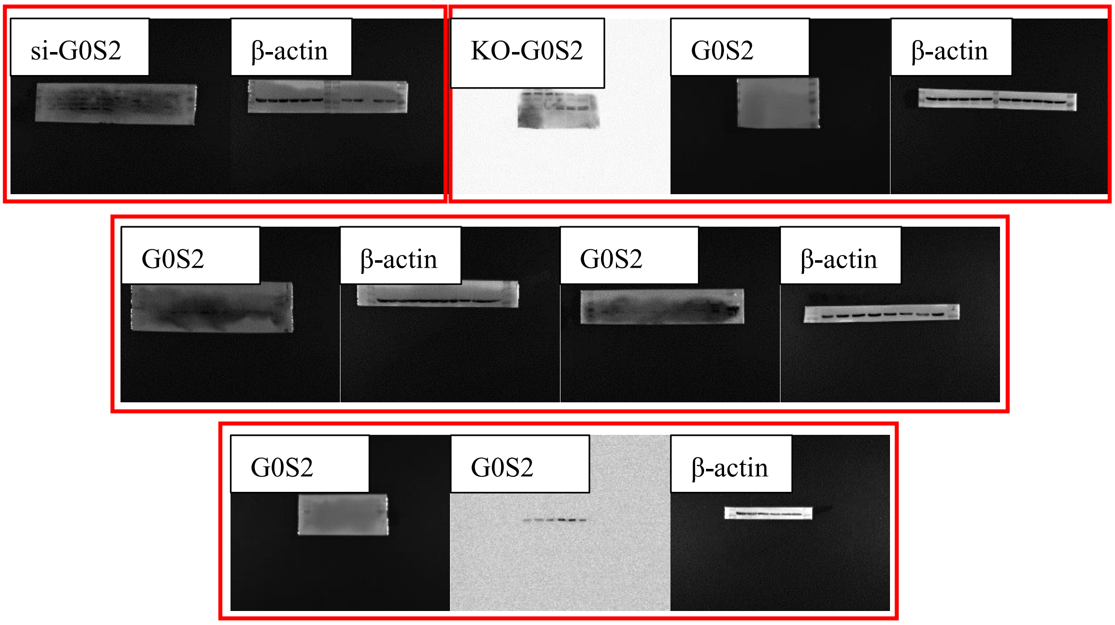

Supplementary Figure 15. Uncropped western blots images for Fig 6

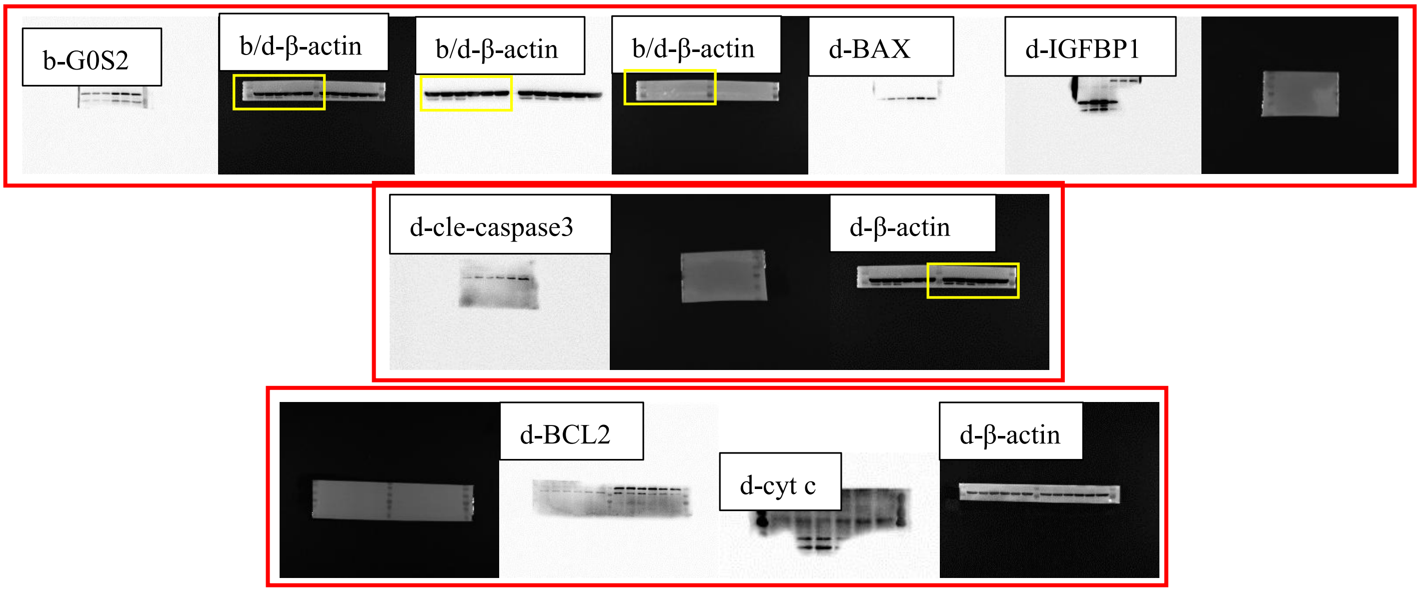

Supplementary Figure 16. Uncropped western blots images for Fig 7

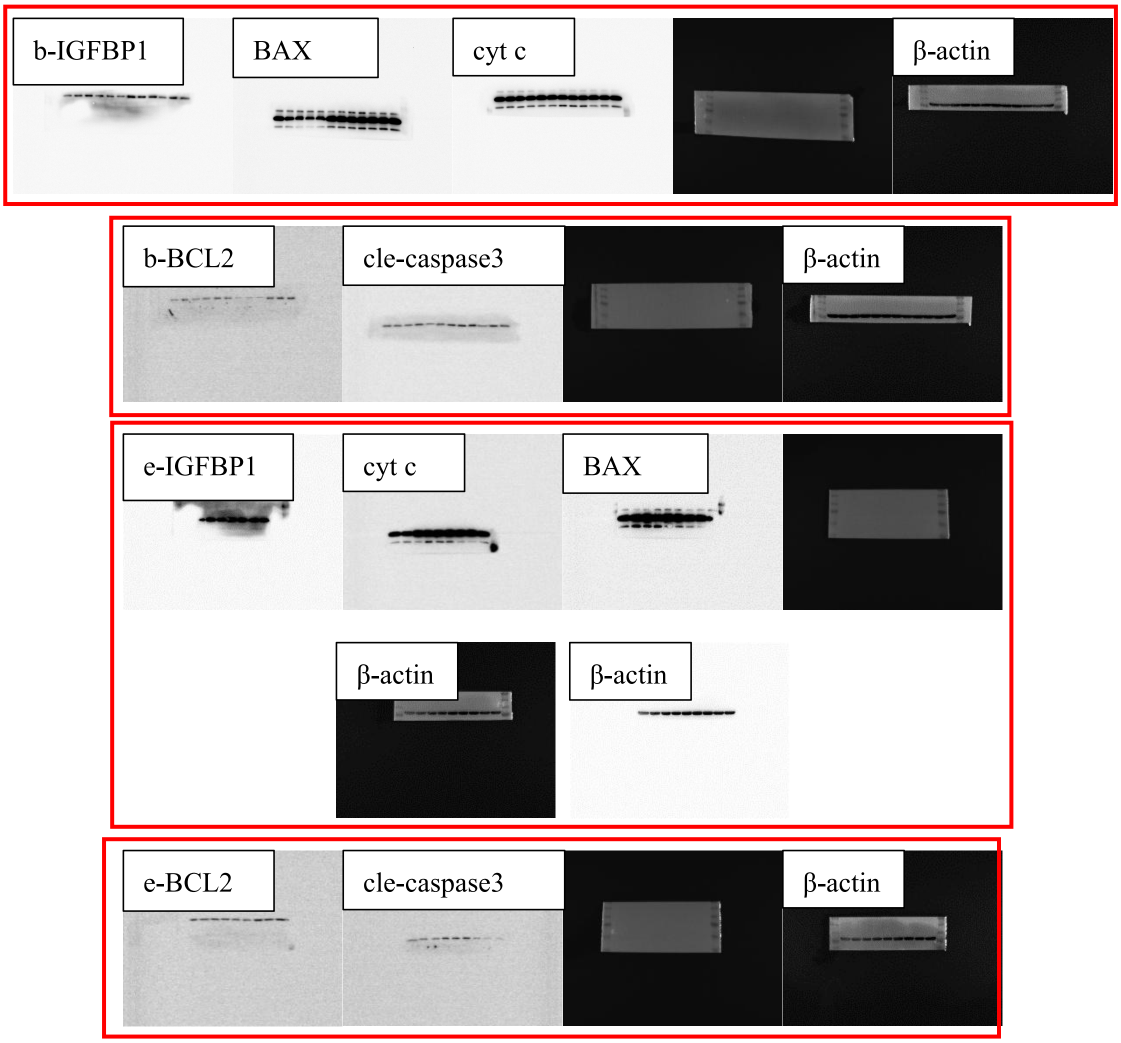

Supplementary Figure 17. Uncropped western blots images for Fig 8

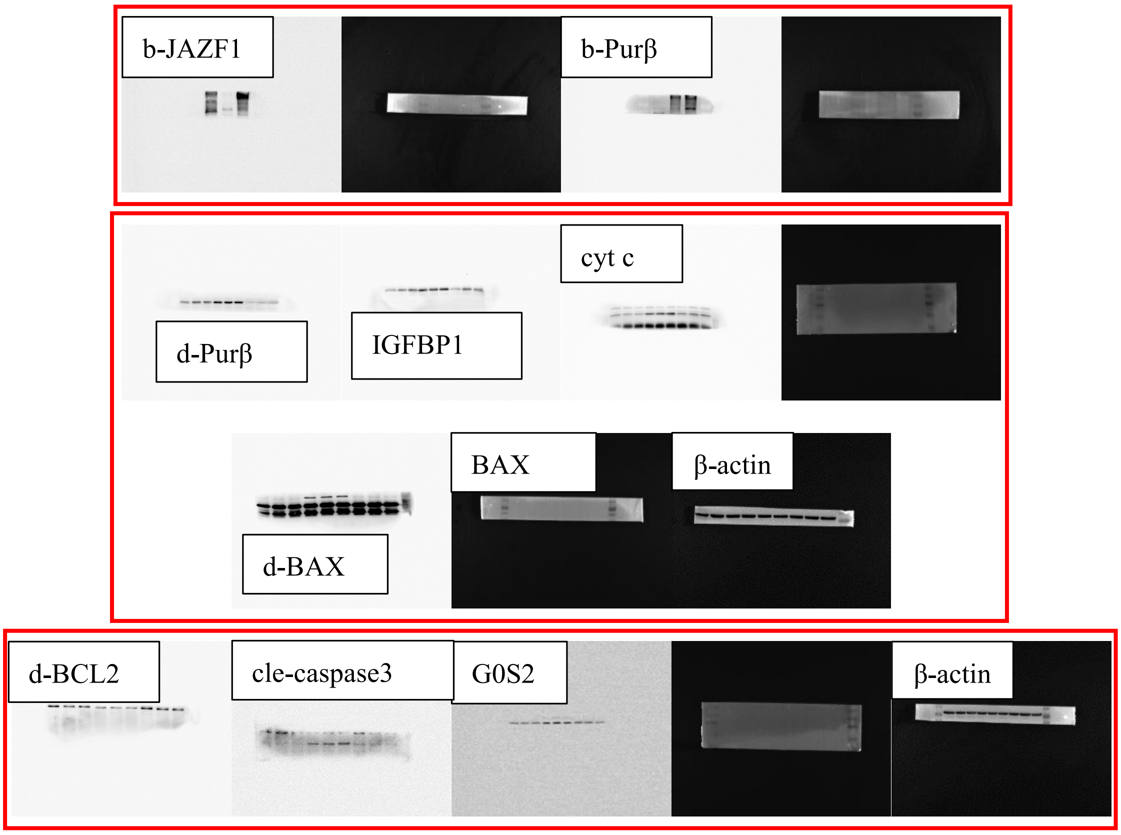

Supplementary Figure 18. Uncropped western blots images for Supp Fig 1

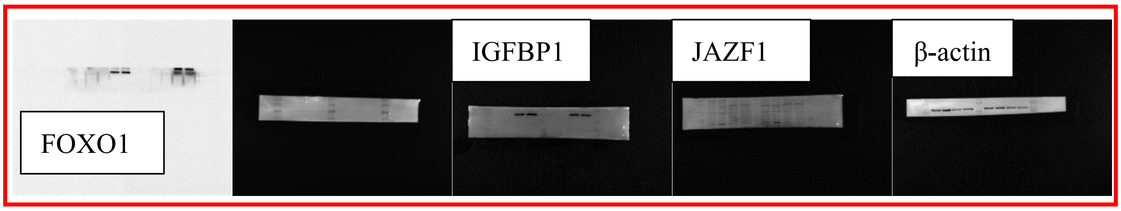

Supplementary Figure 19. Uncropped western blots images for Supp Fig 2

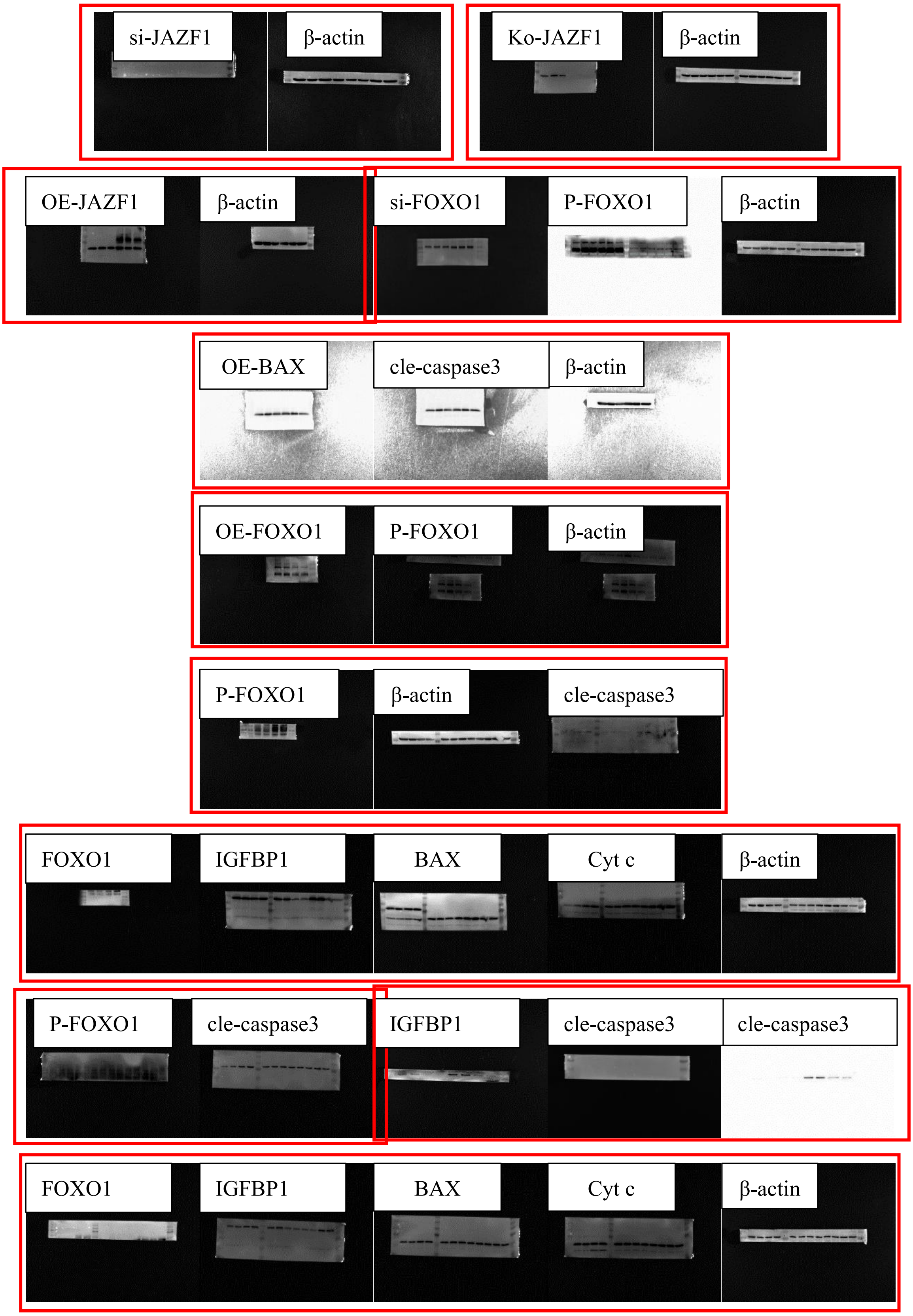

Supplementary Figure 20. Uncropped western blots images for Supp Fig 5

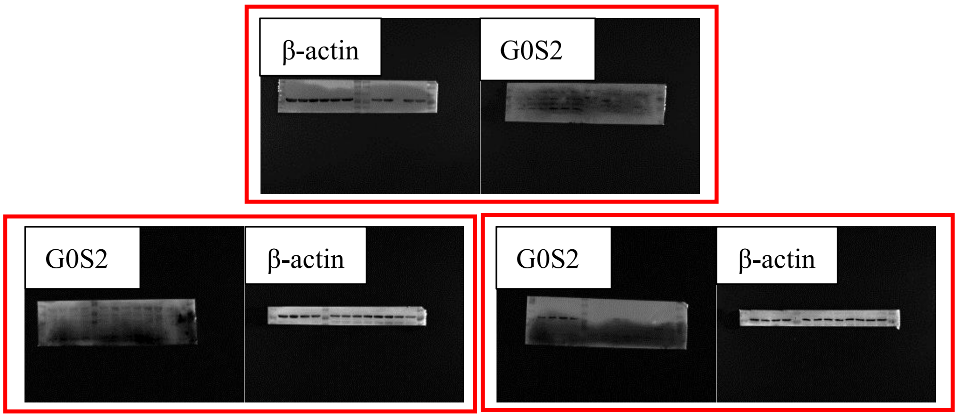

Supplementary Figure 21. Uncropped western blots images for Supp Fig 6

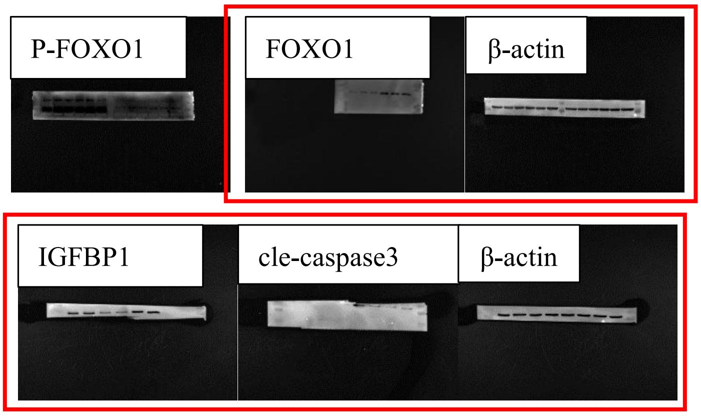

Supplementary Figure 22. Uncropped western blots images for Supp Fig 7

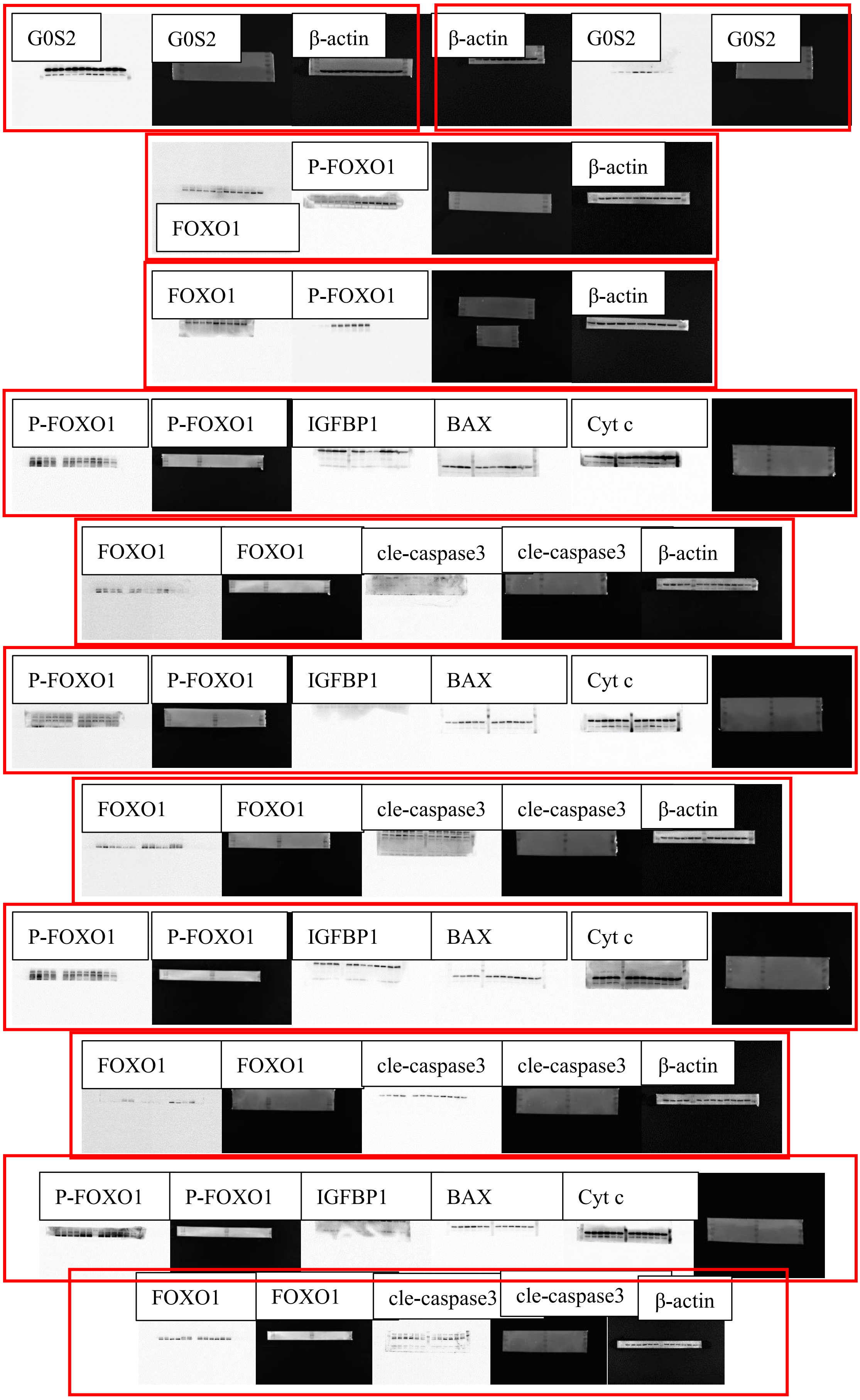

Supplementary Figure 23. Uncropped western blots images for Supp Fig 8

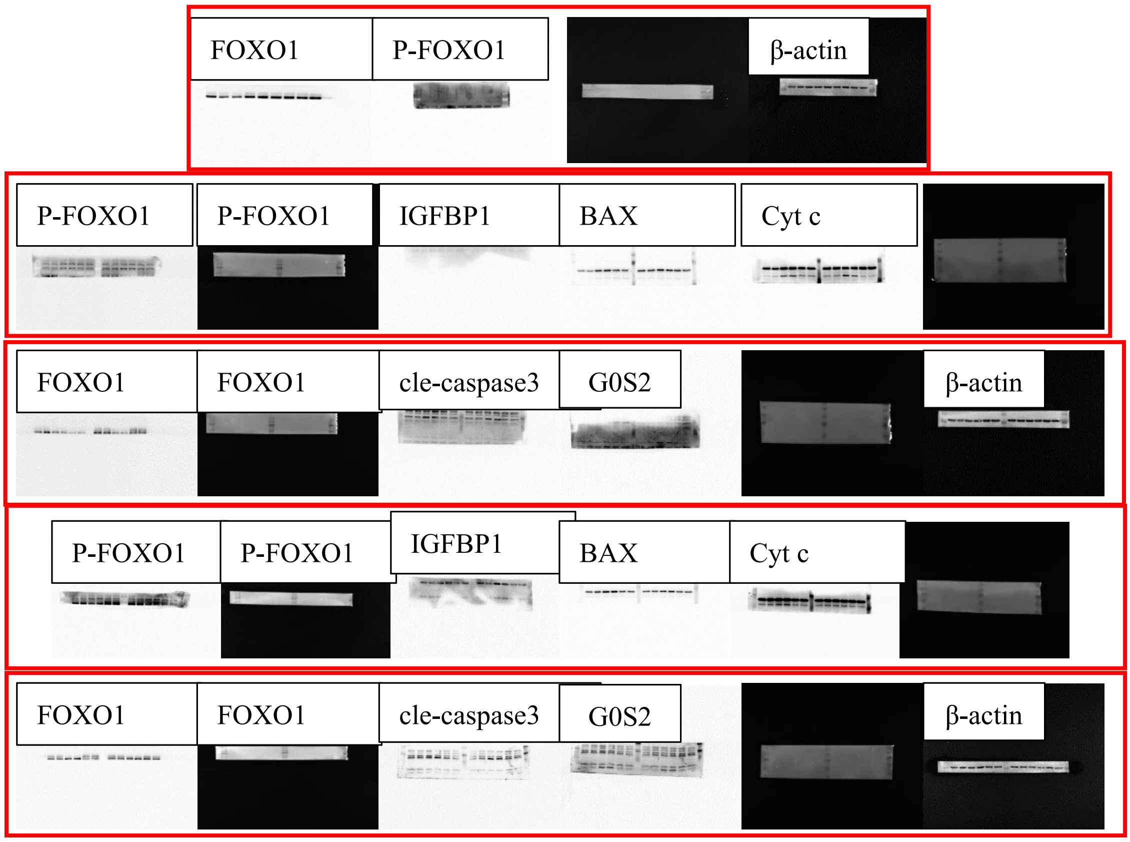

Supplement: Supplementary file 2 — Supplementary Information [file 42003_2023_4931_MOESM2_ESM.pdf]
