# Supplementary material for: JAZF1 safeguards human endometrial stromal cells survival and decidualization by repressing the transcription of G0S2
Source: Commun Biol. 2023 May 27;6:568. doi: 10.1038/s42003-023-04931-x (PMC10224957; doi:10.1038/s42003-023-04931-x)
Supplement: Supplementary file 3 — Description of Additional Supplementary Data [file 42003_2023_4931_MOESM3_ESM.docx]

**Description of Additional Supplementary Files**

**File name:** Supplementary Data 1

**Description:** The reagents used in this study

**File name:** Supplementary Data 2

**Description:** The detailed results of mass spectrometry

**File name:** Supplementary Data 3

**Description:** The source data behind the graphs in the paper
